# “Nursing home staff’s reasoning about providing palliative care following an educational intervention – a qualitative interview study”

**DOI:** 10.1186/s12912-026-05480-4

**Published:** 2026-10-11

**Authors:** Camilla Ekeblom, Carina Lundh Hagelin, Jane Österlind

**Affiliations:** Department of Health Care Sciences, Marie Cederschiöld University, Box 11189, Stockholm, 100 61 Sweden

**Keywords:** Nursing homes, Palliative care, Older people, Interprofessional team, Educational intervention

## Abstract

**Background:**

Palliative care (PC) is increasingly recognised as an essential component of healthcare, yet access remains unequal, particularly for older people. Nursing homes accommodate older people with complex needs and account for a large proportion of deaths, making PC a central component of care in these settings and underscoring the importance of integrating a PC approach into everyday care. However, staff often lack sufficient training and may perceive PC as limited to end-of-life care rather than as an ongoing approach. Education has been proposed to strengthen PC competence, but little is known about how staff reason about PC following such initiatives. This study aimed to explore nursing home staff’s reasoning about providing PC after participating in an interprofessional educational intervention (IPEI).

**Methods:**

A descriptive qualitative design was used. Eighteen staff members from five nursing homes in Sweden, including registered nurses, assistant nurses, a physician, first-line managers, and PC champions, participated. Data were collected through semi-structured individual interviews conducted between March and April 2024 and analysed using inductive qualitative content analysis.

**Results:**

Three main categories were identified: (1) managing one’s own emotions, thoughts, and attitudes; (2) the significance of relationships, communication, and interactions in care; and (3) navigating norms, assumptions, and organisational structures in everyday PC. These categories reflect how staff reasoned about providing PC at individual, relational, and organisational dimensions.

**Conclusions:**

The findings suggest that staff perceived the IPEI as providing opportunities for personal and professional development. Staff’s reasoning further suggested that alignment across individual, relational, and organisational dimensions is important in supporting the provision of PC. Providing PC was not solely a matter of enhancing staff knowledge; rather, staff’s reasoning suggested that knowledge and conditions for care are shaped through the interplay between staff attitudes, their reasoning about care, relational dynamics, and organisational conditions. When these dimensions align and mutually reinforce one another, favourable conditions for providing PC may be supported. Such conditions may also be supported through interprofessional educational initiatives that combine theoretical knowledge with opportunities for reflection and practical application.

**Clinical trial number:**

Not applicable.

## Background

Palliative care (PC) is increasingly recognised as an essential component of healthcare systems worldwide [1]. However, access to PC is found to be unequal, particularly for older people [2–5]. Globally, nearly 57 million people are estimated to require PC each year, and this is expected to increase as populations age and the prevalence of chronic and life-limiting conditions rises [1]. The concept of PC is defined by the International Association for Hospice and Palliative Care (IAHPC) as an active, holistic care of individuals of all ages with serious health related suffering due to severe illness, particularly towards the end of life, with the goal of improving quality of life for patients, their families and caregivers [6].

Of the approximately 90,000 deaths occurring annually in Sweden, around 70,000 are associated with chronic or life-limiting illnesses and can thus be considered potentially in need of PC [7]. Nursing homes (NHs) account for approximately 40% of all deaths in Sweden [8, 9] and are a residential care option for older people who require round-the-clock support and care, often due to frailty, functional impairment, and/or illness [10]. Admission to a NH is generally offered only when an older person is no longer able to remain at home, even with extensive home-care services [11]. As a result, many older persons living in NHs have extensive care needs, multiple chronic conditions and frailty, all of which frequently give rise to PC needs long before the end of life.

According to The European Association for Palliative Care (EAPC) White Paper, addressing complex needs of frail older people requires an integrated approach that combines palliative, geriatric, and rehabilitative care perspectives [12]. Early integration of PC can improve symptom control, support decision-making, maintain quality of life, and enhance overall well-being throughout the later stages of ageing and illness [13–15]. A PC approach should therefore function as an integral part of everyday care. This is also emphasised in national guidelines in Sweden, which state that a palliative approach should permeate all care provided in these settings and that all staff, regardless of profession, should be competent in identifying and addressing PC needs [7].

In NHs, care is provided by a multidisciplinary team with varied educational backgrounds, with assistant nurses (ANs) and care assistants constituting the largest professional groups [16]. These staff members are present around the clock, and work in collaboration with registered nurses (RNs), physiotherapists and occupational therapists. Physician services are provided through the regional primary care system, either directly or through private but regionally funded medical organisations, typically on a consultative basis [17].

Despite the great need for PC, many staff lack specific education in PC and report limited knowledge regarding the complex needs of older people nearing the end of life [18], and staff often feel unprepared to manage PC [19–22]. Furthermore, within the context of NHs, PC has frequently been perceived as separate from everyday care and is commonly associated solely with the final days or week of life, that is, with dying and death [23–25].

In response to the need to strengthen PC, many organisations have introduced so-called PC champions, intended to support knowledge development and quality improvement [26]. However, the role lacks a standardised description, and both its content and organisational conditions vary across settings. Although often described as a local resource for knowledge sharing and support, there is limited knowledge about how these roles function in practice and their contribution to PC.

Effective teamwork is essential in PC, and continuous, structured education is recommended to ensure that all team members are equipped to identify PC needs and support families [7]. National and international reports consistently emphasise the need to strengthen PC competence through structured and continuous educational initiatives [1, 7, 27], and numerous educational interventions have been implemented within NHs [28–33]. However, there is substantial variation in the design and scope of such interventions [29]. Although numerous educational interventions have been implemented in NHs, most have focused on individual professional groups or selected staff categories rather than the entire NH team. Interventions involving all occupational groups including RNs, ANs, care assistants, physicians, physiotherapists, occupational therapists and first-line managers (FLMs) appear to be limited [29]. As PC requires interprofessional teamwork to respond to the diverse needs of older people living in NHs, such educational approaches are of particular relevance. For this reason, an interprofessional educational intervention (IPEI) was implemented to enhance PC competence among staff at five privately operated NHs in a large metropolitan region in Sweden and was designed to bring together members of the NH team [34]. The present study included staff who participated in the IPEI. A brief overview of the IPEI is provided in Table 1.

**Table 1:** The interprofessional educational intervention (IPEI)

**Aim and context **The interprofessional educational intervention (IPEI) in the “From Thread to Mantle” project aimed to enhance palliative care (PC) competence among staff in nursing homes (NHs) in a metropolitan region of Sweden. The IPEI comprised approximately 12 months of active delivery between January 2023 and March 2024, distributed across a 15-month calendar period due to a planned summer break. It was a collaboration between a private care provider operating ten NHs and a healthcare provider responsible for medical services in approximately 130 NHs in the region. Participating units were selected by senior representatives from both organisations.
**Intervention delivery** The intervention was led by a PC specialist team, consisting of a registered nurse (RN) specialised in PC and a physician specialised in geriatrics and palliative medicine. The team visited each NH every second week. Two core components were implemented: • PC rounds • PC workshops
**PC rounds **The PC rounds aimed to identify PC needs at an early stage. A structured checklist was used, covering areas such as serious illness conversations, goals of care, and family support needs. The discussions resulted in documented care plans.
**PC workshops **The workshops aimed to strengthen knowledge of PC and support interprofessional teamwork through reflection and discussion. Six modules were delivered: • Introduction to PC • Symptom management • Communication and relationships • Family support • Teamwork • End-of-life care
**Participants **The intervention targeted an interprofessional team, including RNs, assistant nurses (ANs), care assistants, physicians, occupational therapists, physiotherapists, and first-line managers (FLMs). Participation was voluntary and based on staff availability.
**Extent of implementation **Each participating unit received 23 PC rounds and 22 workshops during the intervention period.
**Additional component – PC champions **PC champions were appointed at each unit to support the implementation by disseminating knowledge and acting as local resources. A total of 20 staff members were trained as PC champions; they received external training in the form of three full-day sessions in spring 2023 [34].

However, little is known about how NH staff reason about PC after participating in interprofessional educational initiatives involving the NH team. Therefore, the aim of this study was to explore NH staff’s reasoning about providing PC in NHs after participating in an IPEI.

## Methods

### Study design

This study used a descriptive qualitative design, which was considered appropriate as it enables a rich description of participants’ perspectives while remaining close to their own accounts [35].

### Setting

Participants were recruited from the five NHs that had taken part in the IPEI. The NHs accommodated individuals aged 65 and older with significant personal care needs and/or cognitive impairment. The NHs together comprised approximately 270 apartments. These are primarily designed for single occupancy, although some units allow couples to live together. Each facility was overseen by a FLM. The workforce primarily consisted of ANs available 24/7, while RNs were present on site during daytime hours throughout the week and were available on call at other times. Services were provided on site by occupational therapists and physiotherapists on designated days, and each NH was affiliated with a physician who conducted scheduled visits once a week. Telephone access to a physician was available to RNs at all times, every day of the week.

### Recruitment

The Quality Manager and FLMs for the five NHs approved the study. All staff who had participated in PC rounds and/or workshops were invited to participate through invitations displayed in staff areas at the five units. Written information about the study was also distributed during monthly staff meetings by FLMs. Some notified their interest to their FLM, who then forwarded their contact details to the research team, while others contacted the research team directly to receive detailed information about the study. Twenty individuals in total registered their interest in participating in the study and were provided with both verbal and written information. However, two of them later declined participation due to time constraints.

### Participants

A total of 18 staff members participated in the study, of whom 15 were women and three were men, with a mean age of 47 years (range 34–62 years). Among the participants were three FLMs, five RNs, one physician, and nine ANs. Six of the participants were PC champions, either RNs or ANs. Five participants had worked in the organisation between one and five years, seven had worked between six and ten years, and six participants had worked for 11 years or more (Table 2). All participants had participated regularly in both PC rounds and workshops; however, detailed records of individual attendance were not available.

**Table 2:** Participant characteristics

Characteristics	Participants (*n* = 18)
Age, mean, (range)	47 (34–62)
Gender	
Female	15
Male	3
Profession	
Assistant nurse	9
First-line manager	3
Physician	1
Registered nurse	5
Working years within the organisation	
1–5 years	5
6–10 years	7
≥ 11 years	6
Palliative care champion	6

### Data collection

Data for the present study were collected between March and April 2024 through semi-structured individual interviews, using a study-specific interview guide with open-ended questions. The interviews were conducted by the first author (CE) who has extensive experience in discussing PC with patients, family members and colleagues. Participants were given the choice of interview format, resulting in 11 face-to-face interviews and seven conducted via the video conferencing tool Microsoft Teams. The interview guide covered three overarching areas: PC, the role of PC champions, and ethical climate. In the present study, the analysis was confined to two of these areas, while the third (ethical climate) will be analysed and reported separately, as it was considered to require a distinct analytical focus. However, the analysis was guided by the study aim rather than by the structure of the interview guide. Thus, the unit of analysis included all interview material relevant to the aim of the present study, irrespective of where it arose during the interview. Data were included based on relevance to the study aim rather than the interview topic in which they emerged. Examples of questions asked included: “What does palliative care mean to you?”, “What kind of support do you feel you and your colleagues need?” and “What kind of support do you feel you can offer your colleagues?”, with follow-up questions such as “Could you tell me more?”, “Could you describe this further?” and “How did you experience this?”. To capture each participant’s unique experiences, the interviews were conducted in a flexible manner, although all areas were covered.

The interviews were audio recorded and lasted an average of 51 min (range 31–77 min). Shortly after each interview, field notes were written to capture contextual information. All interviews were conducted in Swedish. Although some participants did not have Swedish as their first language, all participants were able to communicate in Swedish, and no interpreter or other linguistic support was required. The interviews were transcribed verbatim in Swedish, and the analysis was conducted on the original Swedish transcripts. Quotations included in this manuscript were translated from Swedish into English by the first author (CE). The translated quotations were subsequently reviewed by the other authors (CLH and JÖ) against the original Swedish transcripts to ensure conceptual equivalence and preserve the intended meaning of the original expressions. The adequacy of the sample was continuously evaluated in relation to information power [36]. The sample was considered to provide sufficient information power due to the relatively specific study aim, the inclusion of participants from several professional roles, the focused post-intervention context, and the depth of the interview material. After 18 interviews, the data were considered sufficiently rich and varied to address the study aim.

### Data analysis

Data were analysed using inductive qualitative content analysis as described by Elo and Kyngäs [37]. The first author (CE) manually and verbatim transcribed all recorded interviews. The analytical process then continued with repeated readings of the entire dataset to achieve an overall understanding and to become immersed in the data. During this phase, notes and headings were written in the margins to capture initial thoughts and reflections. Units of analysis (i.e., segments of text relevant to the study’s aim) were subsequently identified and condensed, meaning that they were shortened while preserving their essential content. These condensed units of analysis were assigned codes that captured their core meaning. Codes reflecting similar content and meaning were grouped into subcategories, which were then organised into broader, generic categories. These generic categories were subsequently synthesised into main categories [37].

The analysis was conducted manually in a matrix format using Microsoft Excel, where units of analysis, their condensed forms, codes, subcategories, and categories were systematically organised. The following example illustrates the analysis process: One participant stated: *“I receive a lot of support because I talk about*,* like*,* that I feel uncertain about this*,* then I say how I think*,* like*,* yes*,* and then I can also get confirmation that yes*,* go ahead with that*,* like*,* how you think*,* so I have received very good feedback when things have happened*,* I have great support*,* I can also get that from these registered nurses here.”* The unit of analysis was condensed and coded as “confirmation”. At the next level, the code was grouped into the subcategory “receiving support from colleagues”, which was further organised into the generic category “collegial interactions”. Ultimately, this contributed to the main category “The significance of relationships, communication, and interactions in care.”

CE had the main responsibility for the analysis and conducted all phases in close collaboration with CLH and JÖ. To enhance the credibility of the findings, CLH and JÖ independently read and coded subsets of the data, comprising nine interviews each to verify consistency and confirm the interpretative process. The interviews were distributed between the CLH and JÖ without any predefined selection criteria. Coding, categorisation, and emerging interpretations were continuously discussed within the research team, allowing different perspectives to be explored and a shared understanding of the material to be developed. Regular meetings were held throughout the analytical process, facilitating continuous discussion and reflection among all three authors and enabling dialogue around alternative interpretations to ensure the trustworthiness and depth of the analysis [38]. The authors moved repeatedly between the developing analysis and the original interview transcripts to ensure that interpretations remained grounded in the data [35]. Throughout the analysis, data that did not immediately fit the emerging categories were revisited and discussed among the authors to ensure that the final categories adequately reflected the breadth and variation of the data. Finally, the emerging categories and their relationships were discussed in several meetings until consensus was reached regarding the final structure and interpretation of the findings. To enhance transparency of the abstraction process, the final analytical structure, consisting of main categories, generic categories, and subcategories, is presented in Table 3.

**Table 3:** Final analytical structure

Subcategories	Generic category	Main category
Awareness of personal attitudes towards dying and death	An emotional preparedness	The importance of managing one’s own emotions, thoughts, and attitudes
Emotional responses to caring for older people at the end of life		
Balancing personal emotions and professional responsibility		
Experiencing meaningfulness in PC	An engaged action	
Confidence in providing PC		
Motivation to engage in PC		
Receiving support from colleagues	Collegial interactions	The significance of relationships, communication, and interactions in care
Creating a sense of security through teamwork		
Trusting relationships within the team		
Building relationships with older people and families	The interaction with older people and their families	
Responding to family members’ needs		
Supporting family acceptance of dying		
Experiencing insufficient time and resources	Leadership, structure, and resources	The ability to navigate prevailing norms, assumptions, and organisational structures in everyday PC
Having opportunities for team meetings and reflection		
Receiving managerial support		
Having the mandate to act	Roles and responsibilities	
Recognising and valuing professional competence		
Valuing the central role of ANs		
Shared values within the care culture	The culture within care for older people	
Expectations influencing care practices		
Perceiving PC as a label		

### Researcher role and reflexivity

The IPEI was initially developed as a clinical practice development initiative within the participating organisations. The first author (CE), a RN specialised in PC, was involved in its design. During implementation, the first author was part of a specialised PC team, together with a physician specialised in palliative medicine and geriatrics, leading PC rounds and facilitating workshops. All participants had previously interacted with the first author through PC rounds and workshops. During the interviews, open-ended questions and follow-up probes were used to encourage participants to reflect on both positive and critical experiences of providing PC after participating in the IPEI, including challenges and differing perspectives. Participants were reminded that there were no right or wrong answers and that both positive and critical perspectives were important.

The implementation of complex interventions in healthcare is not solely a matter of knowledge transfer, but also concerns how relationships, roles, and shared understandings are formed over time [39]. Conducting the study in a practice-near context, with active involvement in the IPEI, provided in-depth contextual understanding of the social processes through which change and learning evolve through interaction between different professions. To address potential bias, ongoing reflexive work was undertaken. The first author documented reflections on her preunderstanding, field experiences, and emerging interpretations in field notes, including observations from rounds and workshops, as well as considerations of how her role might have shaped the data. Analytic rigour was further strengthened through continuous discussions within the research team, in which assumptions and interpretations were critically examined. All authors, all RNs with clinical and research experience in PC and geriatric care, contributed to the analytic process to ensure that the findings extended beyond the first author’s clinical perspective.

### Ethical considerations

The study followed the Declaration of Helsinki [40]. The study was submitted for ethical review; there were no ethical objections, and the study was approved (Dnr. 2023-01070-01). Written permission to carry out the research was obtained from both the Regional Manager and the Quality Manager of the five NHs. Since it can be challenging to answer questions about PC, participants were encouraged to contact the researchers, all RNs specialised in PC, if they had any concerns.

### Findings

The analysis revealed three main categories: (1) The importance of managing one’s own emotions, thoughts, and attitudes; (2) The significance of relationships, communication, and interactions in care; and (3) The ability to navigate prevailing norms, assumptions, and organisational structures in everyday PC. These main categories were built upon generic subcategories.

### The importance of managing one’s own emotions, thoughts, and attitudes

This category captures how staff reasoned about their conditions for providing PC by highlighting the importance of managing their own emotions, thoughts, and attitudes when caring for older people with PC needs. This overarching category is shaped by two generic categories: *an emotional preparedness*, in which staff individually reflect on and relate to dying and death, and *an engaged action*, characterised by a genuine interest and internal motivation to provide PC.

### An emotional preparedness

Following the IPEI, staff described the importance of having reflected on their own attitudes towards dying and death to provide PC. They emphasised that they perceived the IPEI to have contributed to their engagement with these issues and to a greater personal acceptance of dying and death. In the care of older people and in encounters with their families, staff described that their work was facilitated when they were able to accept dying and death as a natural part of both everyday work and life more broadly. While some staff reflected on having long perceived dying and death as natural phenomena and therefore not having experienced fear when encountering them in their work, others reflected on perceived changes in their attitudes following the IPEI. These staff spoke of moving from perceiving dying and death as distressing and frightening to experiencing them as more acceptable and manageable. One AN reflected on how the IPEI had contributed to this change:

*It felt a bit terrible at first*,* but now*,* because in the education and workshops we have become used to it*,* we get used to thinking like that… I can manage the grief and the event*,* death*,* now; now it is a normal process for me*,* but in the beginning*,* it was a shock and it was a bit difficult to accept… It was a bit difficult for me*,* but now I know why.* (AN 6)

At the same time, staff described that it could still be emotionally challenging to shift the focus from primarily striving to prolong life to a PC approach with an increased emphasis on comfort, quality of life, and symptom relief. They perceived that some colleagues continued to experience difficulties in accepting dying and death as a natural part of life and everyday work, and that a lack of confidence in dealing with dying and death may lead to a tendency to transfer older persons to acute hospitals in the event of deterioration or to initiate nutritional support when they have stopped eating. Although staff at times found it difficult to let go and accept that the older person they were caring for would not live indefinitely, they emphasised the importance of being able to manage their own emotions when caring for people with PC needs. Some staff described having previously found it difficult to enter the rooms of older people who were approaching the end of life, as this could be emotionally overwhelming. This was partly related to fears of handling a deceased body, which were experienced either personally by the staff themselves or indirectly through colleagues who expressed such fears.

Despite the sadness and emotional burden involved in caring for older people at the end of life with whom close relationships had been developed, staff emphasised the importance of maintaining professionalism to provide PC. One AN described this as:

*It is emotional to work with people*,* I think it can be hard*,* but I still try to keep my professionalism and sort of stay strong when I am here*,* even though it feels difficult inside.* (AN 15)

### An engaged action

The staff perceived it as important to have a genuine interest in PC and an inner motivation to provide good care at the end of life, regardless of whether the older person had years or only hours left to live. Experiencing that, as staff, they could enhance an older person’s quality of life towards the end of life, regardless of the length of this period, and feeling that they had provided the best possible care were described as central conditions for being able to deliver PC following the IPEI. It was also important for staff that PC was not perceived merely as a work task, but as a meaningful part of their professional role and something they could stand for in their work. At the same time, there was a perception that if an individual lacked a genuine interest or inner motivation to provide PC, the conditions for delivering such care well were diminished. As one RN described it:

*Most of them [the ANs] really want to do that little extra*,* they want the residents on the ward to be well… But then there are those*,* I mean*,* there is a shortage [of staff]*,* so then a pair of hands comes in and does what they are supposed to do*,* but you might not have that care*,* you don’t straighten the jumpers or do that little extra*,* and then maybe it does not mean as much to them if something happens*,* because then you are not here for the residents’ sake…* (RN 5)

Having confidence in one’s own competence and clinical assessments was perceived as an important condition for being able to provide PC. Both FLMs and RNs described that ANs appeared to view their role in PC differently following the IPEI. According to these participants, ANs appeared to feel more valued and to have developed a greater understanding of the significance of their role and contributions to care. ANs themselves described perceiving an increased confidence in their competence following the IPEI, although they also described having trusted their own competence and judgement even before the IPEI. One AN expressed their confidence in their own competence as:

*There are people [staff] who get scared when relatives become worried and ask why Mum is lying like this*,* why Mum is in pain. I can’t just walk away and call the nurse*,* I have to sit with her [the relative]*,* calm her down first*,* I also have my profession*,* I also have to talk to her*,* sit with her for a while. If I just show her that I don’t want to talk and I walk away*,* that I feel insecure*,* how will she herself feel safe with me? I want to show her that I am professional in this job*,* so that she feels safe.* (AN 7)

### The significance of relationships, communication, and interactions in care

This main category captures how staff reasoned about the conditions for providing PC through support and communication within the team and in relation to older people and their families. This overarching category is shaped by two generic categories: *collegial interactions*, in which shared communication and mutual support enable staff to manage PC situations, and by *the interaction with older people and their families*, characterised by building trust and relational engagement.

#### Collegial interactions

Feeling both emotional and practical support from colleagues was experienced as an important condition for staff in being able to provide PC following the IPEI. In addition to receiving support, it was also considered important to be able to provide support to colleagues, particularly those with limited experience of caring for older persons with PC needs and their families. Staff experienced that both giving and asking for support was possible, regardless of whether one was a PC champion or not. At the same time, many of those who were not PC champions lacked knowledge about what the role entailed, and it was not always clearly communicated who held this role within the units. There was an understanding among staff regarding colleagues who avoided certain work tasks related to PC, and it was considered important in such situations to provide support, for example by taking over the care of the older person at the end of life or caring for the body after death. Support could also involve sharing knowledge as a way of increasing colleagues’ sense of security and encouraging them to dare to approach dying and death. As one RN described it:

*It could be that you ask: have you been in with this patient? ‘No*,* I haven’t.’ ‘Would you like us to do it together?’ ‘Yes*,* please.’ An example is that you might not really want to go in and ‘no*,* but now the relatives are there.’ ‘Yes*,* and that’s exactly why we perhaps need to be even more visible*,*’ that you get to help them a bit there.* (RN 8)

It was perceived as important that the team could communicate clearly with each other and that different professionals were able to raise issues or express when something did not feel right or felt difficult to understand. Being able as a team to together identify PC needs at an early stage was experienced as facilitating the conditions for providing PC. One of the FLMs said:

*We had a man who was brought up at a palliative care round last spring*,* and he did not die until this autumn*,* so it wasn’t that he was in the final stage of life. And then we brought him up at the palliative care round and discussed different aspects*,* and then I felt that we got a better ending when the time eventually came. Because then you had*,* sort of*,* talked it through beforehand*,* the team was*,* sort of*,* in agreement about what is important*,* what we should think about. Then it didn’t become*,* sort of*,* an abrupt ending. The whole work group thinks about different aspects over a longer period of time*,* which makes the ending better both for the resident*,* for the team*,* and also for the relatives.* (FLM 3)

When staff felt that they did not receive the support they desired, a sense arose that the conditions for providing PC were deteriorating. Not being seen or listened to when expressing need for support was described as lonely and exclusionary. When staff felt that communication was not fully functioning, there was a feeling that this not only affected older people with PC needs but also impacted the staff. One AN expressed it as:

*A nurse could come up and check what the situation was*,* they could maybe give just five minutes*,* but they say that my time is up*,* but if you work with people then you have to show a bit of consideration… I don’t know*,* maybe they could say; give him something calming… they haven’t said that… they haven’t checked what situation I’m in*,* what I’m describing… they just said that it will be fine*,* but it won’t be fine.* (AN 13)

Staff reflected that it was important to trust their colleagues, and this was perceived as an important condition for being able to provide PC, both when they were not present themselves and when work tasks needed to be handed over to someone else. It was important to experience that one’s colleagues had good competence in PC, knew what needed to be done, and took the tasks seriously. However, staff did not always feel trust and a sense of security with their colleagues. As one RN described it:

*Then the evening comes and I watch a bit of the news*,* and then it’s like I start thinking; are they doing the right thing now*,* will the on-call team be able to give this injection? When I have noticed that this isn’t going to work*,* then I have gone out in the evening and helped with the injections and things like that*,* maybe that isn’t very professional*,* but I have done it because I want it to be a good ending. I have been able to work out that there are weak links here during the evening*,* and then there isn’t much sense of security.* (RN 8)

#### The interaction with older people and their families

Staff described that the conditions for being able to provide PC following the IPEI were closely linked to their ability to create and develop relationships and interactions with the older person and the family. Building relationships with both older people and their families was considered significant, and early and continuous contact was described as an important condition for being able to provide good PC.

Staff also perceived it as important to be able to adapt conversations based on where the person was in their process, and it could be easier to talk with those who initiated conversations themselves or clearly expressed a wish to talk. Meeting both the older person and the family required the ability to build relationships and trust, and to gradually deepen conversations as these relationships developed. Staff described that the established relationship they had built could facilitate what they perceived as difficult conversations; at the same time, it could be challenging to maintain a professional role when the relationship had become deeper. It was perceived as important to have the courage to initiate conversations oneself and to dare to ask questions to the older person and the family. One AN expressed it as:

*It’s just that you think more about how you say things and how you do things than you did before. Back then*,* you were more unsure about how to do it. Now it’s almost like you think*,* sort of*,* aha*,* I’ve been doing it right all along*,* but now maybe you dare to ask more about this palliative thing*,* how one might want it to be*,* before they reach that stage*,* like how do you see it*,* or that you ask in a different way… That you dare to ask. Now you can talk more about it than you did before*,* because before the courses and things like that*,* you sort of avoided death itself*,* or the word. Now you can ask*,* how would you like it to be? And then you get these answers in a different way.* (AN 2)

Despite staff describing that the IPEI had contributed to them daring to talk more about PC, providing PC was perceived as challenging when families had difficulty accepting the older person’s situation or taking in information about, for example, a shift in the goals of care. Staff experienced that families’ expectations of care and support sometimes differed from the goals that staff were working towards and what was considered possible to achieve. In these situations, staff described feeling inadequate and finding it difficult to fully meet the needs of families. Staff described that they often experienced families as unwilling to understand or accept the situation, particularly at the end of life. At the same time, early conversations with families were described as facilitating their understanding of what PC entails. Discussing PC at an earlier stage was considered to support families in developing a more nuanced understanding of its purpose, rather than solely associating it with dying, death, or the withdrawal of all medical treatment. As one FLM expressed it:

*The importance of also getting relatives to understand that palliative care is not just what I myself used to perceive it as. When you use the term palliative care*,* people often become very frightened and angry*,* as they feel that it comes so suddenly. I experience that now we use the word earlier*,* which gives the word a different meaning*,* and then it also becomes easier to bring the relatives along in understanding what it is that we can do.* (FLM 3)

Staff experienced that linguistic and cultural differences could affect the conditions for providing support to the older person and to their families. Not sharing the same language could create uncertainty and make it more difficult to take the initiative to start conversations, particularly in situations characterised by grief. At the same time, an AN described how, following the IPEI, they had actively taken steps to overcome these cultural and linguistic barriers:

*Sometimes it becomes difficult*,* you don’t dare to go and talk [with relatives]*,* but now I have taken a step forward myself. Coming from another background*,* the language is a big gap when we talk with them. If you don’t know the language*,* then it is difficult to go forward and talk and grieve or give support… But then I learned that you just go and ask them*,* do you want some coffee or just go in and say hello. *(AN 13)

### The ability to navigate prevailing norms, assumptions, and organisational structures in everyday PC

This main category captures how staff reasoned about the conditions for providing PC within NHs, as shaped by organisational structure and culture. This overarching category is shaped by three generic categories: *leadership*,* structure*,* and resources*, referring to the importance of supportive management, opportunities for team communication and reflection, time and staffing; *roles and responsibilities*, highlighting the importance of clear mandates and role definition within the team; and *the culture within care for older people*, characterised by shared understandings and norms surrounding PC.

### Leadership, structure, and resources

Staff experienced that a key condition for providing PC following the IPEI was having a manager who possessed knowledge of PC and was visible and supportive in practice. It was perceived as important to feel that time was set aside to meet as a team and that there was a structure for sitting down and talking together about the PC needs of older persons and their families. Opportunities for reflection after a person had died were perceived as contributing to conditions that enabled the team to feel proud of the care they had provided. It was important for staff to feel that they had a manager who showed that it was acceptable to prioritise conversations and presence with the older person and the family, and who recognised it as an important part of care. However, staff often experienced that the time was limited, and there was a feeling of not being able to manage everything due to the staffing situation and that other work tasks often had to be prioritised, which could affect the conditions for providing PC in the way they wished:

*You feel that you don’t have time for all of that*,* sort of… and still you have to stay calm*,* you have to answer all those questions*,* you can’t say; you know*,* others are waiting for me*,* I can’t say that… but sometimes I have no possibility.* (RN 17)

### Roles and responsibilities

The role of ANs was described as one of the most important for being able to provide PC following the IPEI. At the same time, there was a perception that the competence of ANs was not always fully utilised. They were described as those who worked closest to the older person and their family, and who most often developed close relationships and had a good knowledge of the individuals they encountered. ANs described that they often had confidence in their competence but did not always know whether it was acceptable to express the information they had, or whether it was acceptable to engage in deeper conversations about, for example dying and death. An important condition highlighted by staff following the IPEI was the need to clarify the role of the AN and to ensure that the entire team recognised that the role carried a clear mandate:

*“Care staff are the ones who see the resident and the relatives the most*,* but when residents mention death*,* then suddenly someone with a university degree has to come in to talk about it*,* and I really don’t think that’s necessary at all. I think the care staff can do it.”* (Physician).

The role of the PC champion did not emerge as specific or clearly defined; rather there was a perception, both among the champions themselves and among other staff, that the champions had not quite found their place following the IPEI.

### The culture within care for older people

Staff described how communication within the NHs was important for providing PC following the IPEI. When PC was clearly expressed as a natural and integrated part of care within the NH, staff perceived it as easier to provide PC. This was experienced as facilitating collaboration within the team and in meetings with the older person and their family:

*It sounds so pretentious to say that it is an honour*,* but in the end it actually is. I usually joke with the older persons who sit here at the first meeting with me and say that you’ve had a long life*,* you’ve taken care of children*,* grandchildren*,* family*,* home*,* had a job*,* and now it’s time to check in*,* all inclusive*,* we take care of everything*,* now it’s your turn to enjoy and just have a lovely final time… and that is some kind of vision that we want to spread to the older persons who come to us*,* that here you can feel safe*,* we have the knowledge needed to take care of you until your very last breath.* (FLM 1)

At the same time, staff described that it sometimes could be experienced that the image of NHs conflicted with how PC ought to function. Staff experienced that they actively needed to reflect on how they talked about PC in order not to reinforce misleading perceptions of what PC did and did not involve. The very concept of PC was described as carrying a “label” that could negatively affect the conditions for providing PC, as it was often associated with no longer do active interventions. This could make it more difficult to address early PC needs, both within the team and in encounters with the older person and their family. When descriptions of NHs conflicted with staff’s understanding of PC, the conditions for openly talking about death and dying were perceived to deteriorate. As one AN described it:

*This clashes with the culture and purpose of elderly care*,* we give them the feeling that it’s a hotel that mum or dad is moving into*,* so then it’s a bit difficult to convey that kind of information to them… we think that you don’t die in a hotel… so that part is actually a bit difficult…* (AN 6)

Staff experienced that a lack of knowledge about PC was still widespread and that old perceptions of PC persisted, both within NHs and in society, which was perceived as affecting the conditions for applying new knowledge in practice. When the concept of PC continued to be associated with the very final phase of life or with “not doing anything more”, it was experienced as difficult to create a shared understanding of and communicate an early and integrated PC approach:

*I can feel that the lack of knowledge is very great regarding the fact that palliative care starts early. Even though we had taken the course*,* there was still someone*,* a nurse even*,* who says; yes*,* but she isn’t palliative*,* she walks around and eats and talks*,* she’s not palliative… then it becomes extremely difficult to convey knowledge… I can feel like*,* oh*,* what a job this is*,* it’s like walking into a wall sometimes.* (RN 18)

## Discussion

The present study explores how NH staff, after participating in an IPEI, reasoned about providing PC as an individual, relational, and organisational matter, and how these aspects influenced their perceived conditions for providing PC. Staff perceived that the IPEI contributed to their possibilities for providing PC. The findings further suggest that individual, relational and organisational conditions were closely interrelated. Individual aspects were perceived as being shaped through collegial interactions and interactions with older people and their families, while relational aspects were in turn influenced by individuals within the team. Furthermore, both individual and relational aspects were affected by organisational conditions and workplace culture. The IPEI provided a structure in which staff regularly engaged in PC rounds, workshops and interprofessional discussions. While no conclusions can be drawn regarding intervention effects, this structure may help explain why staff highlighted issues related to reflection, collaboration, professional roles and organisational conditions when reasoning about PC in a post-intervention context.

Staff in the present study reasoned that conditions for providing PC were facilitated when those delivering care demonstrated emotional preparedness and engaged action. It became easier to provide PC when staff accepted the inevitability of death as a natural part of life, a finding also reported by others [41, 42]. A sense of committed engagement in delivering PC was further described by staff as shaping their ability to provide such care. Previous findings have shown that a lack of interest among staff may constitute a significant barrier to providing PC. For example, approximately 30% of the 679 NH physicians in Norway and the Netherlands reported that their own lack of interest in end-of-life care constituted an important barrier [43]. In line with the present findings, this suggests that the level of personal engagement and interest in end-of-life care may influence the extent to which staff engage in and provide PC. While committed staff are widely recognised as essential for delivering PC in NHs [44], previous studies have raised concerns regarding variability in staff willingness to provide such care [45]. This aligns with findings indicating that PC quality is adversely affected when staff lack enthusiasm and genuine interest in their work [42, 46, 47]. Individual attitudes towards death and dying may influence staff members’ willingness to engage in PC, and this relationship may be bidirectional. Engagement in PC practice may, over time, support the development of greater acceptance of death, while discomfort with death may reduce both interest and emotional investment in such care. Previous research indicates that educational interventions can positively influence staff attitudes towards caring for people at the end of life and their families within NHs [31]. Similarly, increased knowledge of PC has been associated with more positive attitudes towards PC for people with advanced dementia [48]. Conversely, limited understanding and negative perceptions of PC among staff have been identified as barriers, contributing to reluctance to assume responsibility for PC provision [29]. The present study adds to previous research by showing how staff reasoned about emotional preparedness and engagement as important conditions for providing PC, and how staff perceived the IPEI as contributing to these conditions. The findings suggest that staff perceived reflection on death and dying, as well as increased confidence in one’s role, as important for acceptance of death and engagement in PC practice.

Staff perceived participation in the IPEI as having contributed to a stronger internal sense of mandate, reflected in an enhanced feeling of “I can”, while simultaneously highlighting the importance of being included on equal terms within the team. The role of ANs was described as crucial for the delivery of PC in NHs. ANs themselves reasoned that they observed much, possessed valuable knowledge, and made important contributions. This view was shared by RNs, physicians, and FLMs, who emphasised the need for ANs to be acknowledged and recognised. A sense of mandate derived from colleagues was considered essential for perceiving the quality of PC as good, underscoring the importance of a supportive team culture in enabling the provision of PC. Insights from earlier research involving NH staff who participated in an educational intervention suggest that increased attention to interprofessional culture, such as mutual recognition, open communication, and reduced hierarchical barriers, may support collaboration and enable staff contributions in PC practice [42]. This aligns with previous research demonstrating that interdisciplinary collaboration, collegial support, and psychological safety, where staff feel able to share responsibility and openly discuss the emotional impact of PC, are key enablers of high-quality PC in NHs [41, 47]. Despite this, ANs in the present study at times expressed that they “were not allowed” to act, which may reflect ongoing ambiguity regarding role boundaries, insufficient collegial support, or norms and values embedded within the organisational culture. These findings suggest that the mandate of ANs needs to be made more visible and reinforced across multiple organisational dimensions.

While interprofessional collaboration and shared reflection emerge as central to the provision of PC, the present findings also illustrate how organisational conditions may hinder such practices. In NHs, where parts of the team are employed on an on-call basis, creating time and continuity for regular team meetings is challenging. This tension between the recognised importance of teamwork and the realities of care organisation warrants further attention when developing and implementing PC IPEIs in this context.

Facilitation has been identified as a key factor in the successful implementation of PC interventions in NHs [49]. In this context, PC champions may be expected to act as internal facilitators, supporting colleagues and helping to translate PC knowledge into everyday practice. However, the findings indicate that the role was not always clearly defined in practice, and that it was not consistently evident who held this function. Previous studies have similarly shown that fulfilling this role is challenging, and that adequate support and clearly defined responsibilities are required for it to function effectively [50]. This raises questions regarding the mandate of PC champions and the organisational conditions required for them to function as sources of support in PC. If the role is not embedded within the organisation, either among colleagues or within leadership structures, opportunities to influence practice, disseminate knowledge, and support colleagues may be limited. Clearer organisational structures may therefore be necessary, including explicit expectations and recognition of the role by management. Given that ANs in the present study at times experienced limited mandate to provide PC, such organisational conditions may also have contributed to ambiguity surrounding the role of the PC champion.

Staff in the present study described working in a context shaped by norms, assumptions and organisational structures that at times constrained their ability to provide PC. This underscores the significance of workplace culture for how PC can be delivered. Even when individuals and teams perceived that participation in the IPEI had supported more favourable conditions for providing PC, these efforts could be constrained by the persistence of traditional views of PC within NHs and society more broadly. Staff described how the everyday logic of care provision in NHs sometimes conflicts with a PC approach. This tension may be reinforced by a prevailing belief that PC is primarily relevant only in the final days of life, rather than as a complementary approach throughout the illness trajectory [51–53]. Such perceptions highlight that cultural change within NHs, not merely individual competence development, is essential to enable PC provision. Indeed, across 13 studies conducted between 2003 and 2019, including studies from Sweden, public knowledge and awareness of PC were consistently low, with PC mainly associated with terminal illness and end-of-life care [52]. This limited understanding is likely to shape expectations and decision-making, thereby restricting opportunities for timely access to and delivery of PC within NHs. Therefore, it is essential to communicate that PC is relevant not only at the very end of life but throughout the course of illness, both in conversations with people with PC needs and their families and among the staff providing their care [7].

The present study illustrates how staff perceived the IPEI as contributing not only to their knowledge, but also their understanding of their professional roles, their ways of relating to one another, and their navigation of organisational structures. Staff further described greater awareness of their own views on life, death, and PC, which they perceived as important in shaping their approach to providing PC. Previous research suggests that educational initiatives often strengthen staff members’ self-efficacy, confidence and motivation to provide PC; however, such interventions do not necessarily translate into improvements in older people’s quality of life or quality of death in NHs [54]. Previous research also underscores that educational initiatives need to be followed by ongoing support and follow-up in order for newly acquired knowledge to be implemented, embodied in practice, sustained over time, and made meaningful in everyday care [55]. This discrepancy may be explained by relational and organisational conditions that shape staff members’ ability to apply newly acquired knowledge in practice. Earlier studies have shown that a lack of continuity constitutes a barrier to providing PC for people with severe dementia in NHs [56]. Time pressure and increasing efficiency demands further restrict staff members’ capacity to provide PC, while the frequent use of temporary staff undermines continuity in both care planning and delivery [42, 56]. Furthermore, even when permanent staff participate in educational interventions, NHs often rely on temporary staff who may not be afforded the same opportunities for training, resulting in persistent knowledge gaps that may continue to hinder the provision of PC. Although staff perceived that participation in the IPEI contributed to increased knowledge and motivation among staff, individuals who had not taken part in the training continued to influence the care environment, highlighting a perceived gap between individual, relational and organisational conditions and the need for recurring training.

Previous studies have similarly shown that organisational culture and insufficient managerial support limit NHs’ ability to provide PC [29], which may help explain why changes described by staff were contingent upon relational and organisational structure. This may be interpreted as an indication that more profound changes in the care and organisational culture within NHs are required. Persson et al. [42] have also problematised this issue, and previous research demonstrates that many barriers to change are not solely related to a lack of knowledge or individual working practices but are instead deeply embedded in the local organisational culture, which makes them difficult to change in the short term [57]. This is consistent with findings by Möllerberg et al. [58], who similarly argue that the successful implementation of PC approaches extends beyond individual effort and isolated assessment tools. Achieving a NH culture in which values, norms, and assumptions are aligned with evidence-based PC presents a significant and complex challenge [46]. The findings suggest that opportunities for interprofessional reflection and discussion were perceived as important by staff and may be integrated into everyday practice in NHs. In addition, relational and organisational conditions, including collaboration, role clarity, and managerial support, may be important considerations in the design of initiatives intended to support PC in NHs.

Taken together, these findings point to the need for further exploration of the ethical dimensions of providing PC in NHs, particularly how staff members reason about their responsibilities, engage in ethical reflection, and navigate hierarchical structures in situations where their ability to act in accordance with their professional judgement is constrained. Such knowledge may contribute to a deeper understanding of the ethical challenges inherent in everyday PC practice in NHs.

### Strengths and limitations

Only one physician participated in the study, and no physiotherapists or occupational therapists, which restricts the range of professional perspectives represented. The findings therefore primarily reflect the experiences of ANs, which should be considered when interpreting the findings. At the same time, this distribution largely reflects the staffing composition typically found in NHs, where ANs constitute the majority of the workforce, and may therefore provide a realistic picture of everyday practice. Although all participants had participated regularly in the PC rounds and workshops included in the IPEI, variation in the extent of participation could not be quantified. Consequently, participants may have been exposed to different amounts of the intervention, which should be considered when interpreting the findings. The inclusion of staff whose first language was not Swedish can also be understood as both a strength and a potential limitation. While it may have affected the depth and nuance of some responses, it enabled the inclusion of perspectives that are often underrepresented in research and reflects the diversity of the workforce in many NHs. Participant recruitment was based on voluntary participation, and it cannot be ruled out that individuals with a particular interest in PC or more positive experiences of the intervention were more inclined to take part. Although participation was voluntary, some participants initially expressed interest through their FLM, which may have influenced participation decisions for some individuals. However, the data indicate that participants reflected not only on aspects that functioned well, but also on challenges and difficulties related to providing PC. The first author’s involvement in both the intervention and the data collection may also have increased the risk of social desirability bias, as some participants may have been reluctant to express criticism of an intervention in which the interviewer had been involved. At the same time, this involvement may be viewed from multiple perspectives. While it could have influenced participants’ willingness to express criticism, it may also have facilitated openness, as prior familiarity and trust can support more in-depth and nuanced accounts. Moreover, the researcher’s contextual knowledge of the setting may be considered a strength, enabling richer interpretation of the data.

## Conclusions

Taken together, these findings suggest that staff perceived the IPEI as providing opportunities for personal and professional development, while effective implementation of a PC approach in practice requires alignment across individual, relational and organisational dimensions. Providing PC for older people living in NHs is not solely a matter of enhancing staff knowledge. Rather, staff’s reasoning suggested that knowledge of PC and the conditions for providing PC are shaped through the interplay between staff attitudes, reasoning about care, relational dynamics, and organisational structures. When these dimensions align and mutually reinforce one another, favourable conditions for providing PC may be supported. Staff’s reasoning suggested that such conditions may also be supported through interprofessional educational initiatives that combine theoretical knowledge with opportunities to reflect on and develop staff’s reasoning in relation to practical application.

## Data Availability

The datasets generated and/or analysed during the current study are not publicly available due to the sensitive nature of the qualitative interview data and the need to ensure participant confidentiality but are available from the corresponding author on reasonable request.

## Abbreviations

AN: Assistant nurse
FLM: First–line manager
IPEI: Interprofessional educational intervention
NH: Nursing home
PC: Palliative care
RN: Registered nurse
